# Adipogenesis: A Complex Interplay of Multiple Molecular Determinants and Pathways

**DOI:** 10.3390/ijms21124283

**Published:** 2020-06-16

**Authors:** Melvin A. Ambele, Priyanka Dhanraj, Rachel Giles, Michael S. Pepper

**Affiliations:** 1 Department of Immunology, and SAMRC Extramural Unit for Stem Cell Research and Therapy, Institute for Cellular and Molecular Medicine, Faculty of Health Sciences, University of Pretoria, Pretoria 0001, South Africa; melvin.ambele@up.ac.za (M.A.A.); priyanka.dhanraj162@gmail.com (P.D.); rachel26364@gmail.com (R.G.); 2 Department of Oral Pathology and Oral Biology, School of Dentistry, Faculty of Health Sciences, University of Pretoria, Pretoria 0001, South Africa

**Keywords:** adipogenesis, adipocyte commitment, adipocyte progenitor, transcription factor, miRNA, signalling pathway, epigenetic regulator, adipose tissue

## Abstract

The formation of adipocytes during embryogenesis has been largely understudied. However, preadipocytes appear to originate from multipotent mesenchymal stromal/stem cells which migrate from the mesoderm to their anatomical localization. Most studies on adipocyte formation (adipogenesis) have used preadipocytes derived from adult stem/stromal cells. Adipogenesis consists of two phases, namely commitment and terminal differentiation. This review discusses the role of signalling pathways, epigenetic modifiers, and transcription factors in preadipocyte commitment and differentiation into mature adipocytes, as well as limitations in our understanding of these processes. To date, a limited number of transcription factors, genes and signalling pathways have been described to regulate preadipocyte commitment. One reason could be that most studies on adipogenesis have used preadipocytes already committed to the adipogenic lineage, which are therefore not suitable for studying preadipocyte commitment. Conversely, over a dozen molecular players including transcription factors, genes, signalling pathways, epigenetic regulators, and microRNAs have been described to be involved in the differentiation of preadipocytes to adipocytes; however, only peroxisome proliferator-activated receptor gamma has proven to be clinically relevant. A detailed understanding of how the molecular players underpinning adipogenesis relate to adipose tissue function could provide new therapeutic approaches for addressing obesity without compromising adipose tissue function.

## 1. Introduction

A 2020 World Health Organization report states that globally, 39% and 13% of adults 18 years and older are overweight and obese, respectively. It also lists obesity as a chronic disease that has nearly tripled since 1975 [1]. Obesity is a risk factor for many non-communicable diseases such as type 2 diabetes, cardiovascular diseases and hypertension, respiratory disorders, certain cancers, and various other diseases and disabilities [2,3]. The aetiology of obesity is multifactorial and involves an interaction between genetic and environmental factors [4,5]. Environmental factors have played a major role in the dramatic increase in the global prevalence of obesity. Several studies have shown that obesity results from an imbalance between energy consumed and energy spent [3,6,7]. Diets high in saturated fats, sugar, and processed foods increase caloric intake. This, together with reduced physical activity, results in an energy imbalance [6,7]. Excess energy is stored as lipids in adipocytes either through the process of adipocyte hyperplasia (formation of new adipocytes) or hypertrophy (enlargement of existing adipocytes). These two mechanisms result in increased fat mass and increased secretion of fatty acids, peptides, inflammatory cytokines, and adipokines [3,8].

Adipose tissue is a loose connective tissue found beneath the skin (subcutaneous) and/or surrounding organs and tissues, and it contains a collection of mature adipocytes, preadipocytes, mesenchymal stromal/stem cells (MSCs), vascular endothelial and contractile cells (pericytes and smooth muscle cells), nerves, and an array of immune cells [9]. Adipose tissue is essential for the regulation of energy supply and acts as a caloric reservoir [5]. Adipose tissue is also an active endocrine organ that secretes numerous bioactive peptides and proteins that play a role in controlling and maintaining the activity of other cells and organs. These adipocyte-secreted factors are collectively referred to as adipokines and include cytokines, hormones, growth factors, and acute phase proteins [2]. There are two main types of adipose tissue, brown adipose tissue (BAT) and white adipose tissue (WAT).

Adipocytes that constitute BAT have distinctive features such as the presence of many intracellular lipid droplets and numerous mitochondria, as well as high levels of expression of mitochondrial uncoupling protein 1 (UCP1), that distinguish it from WAT. The main function of BAT is non-shivering thermogenesis in response to cold stress or β-adrenergic stimulus [10]. Activated BAT takes up fatty acids and glucose to provide fuel for sustained thermogenesis [11]. The cells that constitute BAT secrete adipokines such as fibroblast growth factor 21 (FGF21), interleukin 6 (IL6), and chemerin; it is not clear however whether BAT performs functions other than regulating thermogenesis [12,13]. The distribution of BAT in mice and humans varies. In mice, large BAT depots are located in the inter-scapular, subscapular, and cervical regions, while smaller depots are found around the aorta and in the hilum of kidney [14]. Analysis of glucose uptake in humans by ^18^fluoro-2-deoxy-d-glucose position emission tomography-computed tomography (^18^FDG PET-CT) showed that the location of BAT is not limited to the carotid artery, aorta and subscapular region, but that it is widespread and inversely proportional to body mass index (BMI) [15,16].

White adipose tissue is the most abundant type of adipose tissue composed of adipocytes characterized by a large unilocular lipid droplet whose main function is energy storage. White adipose tissue is also secretes adipokines like leptin and adiponectin for energy homeostasis [16]. Anatomically, WAT depots can be classified as being either subcutaneous or visceral [17]. Studies in mice mostly make use of inguinal subcutaneous WAT (scWAT) and perigonadal visceral WAT (in male mice) to represent subcutaneous and visceral depots, respectively. Depots of scWAT in mice include the interscapular, anterior and posterior inguinal scWAT, while visceral depots are mesenteric, perigonadal, and retroperitoneal [16].

A third class of adipose tissue is located within WAT and is known as brite/beige, containing adipocytes with characteristic features of BAT, being induced by cold stress [18]. Researchers have argued that brite adipocytes within scWAT depots are completely different from resident white adipocytes, and have even suggested the entire inguinal scWAT to be a brite adipocyte organ [19]. The function of brite adipocytes is thermogenesis to maintain body temperature; these cells express levels of UCP1 mRNA similar to brown adipocytes. Brite adipocytes also have a positive effect on whole body glucose regulation, and have been suggested to play a role in the treatment of type 2 diabetes [16].

**Figure 1:**
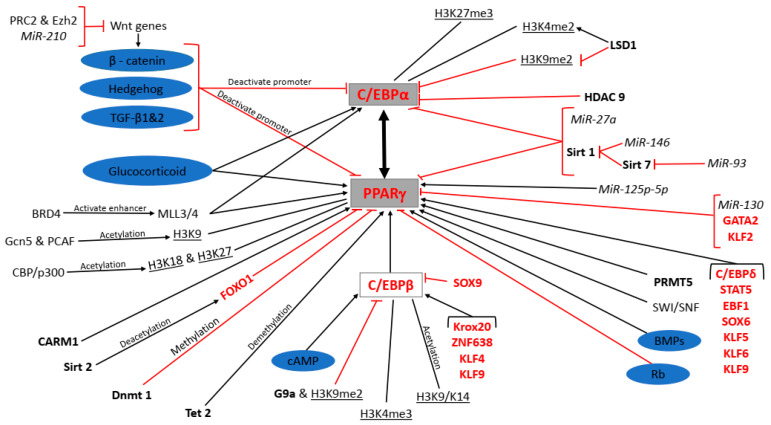
Molecular regulation of peroxisome proliferator-activated receptor gamma (PPARγ) and CCAAT-enhancer-binding protein alpha (C/EBPα) expression during adipogenesis. Signalling pathways are shaded purple, miRNAs in italics, enzymes in bold, transcription factors in red, epigenetic modifications underlined, protein complexes and genes in standard font and the master regulators of terminal differentiation shaded grey.

Adipose tissue develops during embryogenesis in week 14–24 of gestation in humans [20]. Adipocytes are generally believed to originate from multipotent MSCs, precursor cells that arise from the mesoderm. Adipocytes located in the craniofacial region are generated from the neuroectoderm [21]. The development of adipocytes is an ongoing process, continuing throughout an individual’s lifespan. Over the last few years, several studies have investigated the development of adipocytes (adipogenesis) to better understand how new adipocytes are formed and what factors are involved. The molecular mechanisms that direct the differentiation of adipocyte precursor cells (brown or white or brite) into mature adipocytes are complex, and consist of many molecular players. This review will discuss the differentiation of white adipocyte precursor cells (preadipocytes) down the adipogenic lineage, with a focus on some of the key molecular players which include but are not limited to signalling pathways, epigenetic regulators, transcription factors, and others. It will also provide an integrated and interactive synopsis of the molecular players regulating the expression of peroxisome proliferator-activated receptor gamma (PPARγ) and CCAAT-enhancer-binding protein alpha (C/EBPα), which are central to the regulation WAT adipogenesis. A summary of these interactions is shown in Figure 1, which integrates the different categories of molecular regulators.

Signalling pathways such as the canonical Wnt/β-catenin, Hedgehog and transforming growth factor beta (TGF-β) 1 and 2, as well as Sirtuin (Sirt) 1, microRNA (MiR)-27a and MiR-93, inhibit PPARγ and C/EBPα expression. Conversely, the glucocorticoid, cAMP, and bone morphogenetic proteins (BMPs) signalling as well as methyltransferase mixed lineage leukaemia protein 3/4 (MLL3/4) polycomb repressive complex 2 (PRC2) and enhancer of zeste homolog 2 (Ezh2), MiR-210 (MiR-210), bromodomain-containing protein 4 (BRD4), Sirt 7 and MiR-146 promote PPARγ and C/EBPα expression. H3K9me2 and HDAC9 directly inhibit C/EBPα expression while H3K27me3 and H3K4me2 directly promote it. LSD1 promotes and inhibits C/EBPα expression indirectly through H3K4me2 and H3K9me2, respectively. H3K9, H3K18, H3K27, coactivator-associated arginine methyltransferase 1 (CARM1), ten eleven translocation (Tet) 2, C/EBPβ, C/EBPδ, signal transducers and activators of transcription (STAT)5, early B-cell factor 1 (EBF1), sex determining region y-box (SOX) 6, kruppel-like factors (KLF) 5, 6 and 9, MiR125p-5p, switch/sucrose nonfermenting family (SWI/SNF), and protein arginine methyltransferases (PRMT) 5 directly promote PPARγ expression, while general control non-depressible 5 (Gcn5) and p300/CREB-binding protein (CBP)-associated factor (PCAF), CBP/p300, H3K4me3, H3K9/K14, Krox20, zinc finger factor 638 (ZNF638), KLF4, and KLF9 are the indirect promoters of PPARγ expression. Forkhead box protein O1 (FOXO1), DNA methyl transferase (Dnmt) 1, GATA2, KLF2 and MiR-130 directly inhibit PPARγ expression while Sirt 2, G9a and H3K9me2 and SOX9 are the indirect inhibitors of PPARγ expression. PPARγ or C/EBPα transactivate each other.

## 2. Adipocyte Tissue Progenitors

A panel of cell surface markers which includes positive expression of platelet-derived growth factor receptor α (PDGFRα), cluster of differentiation 34 (CD34), CD24, CD29, and spinocerebellar ataxia type 1 (Sca1), and negative expression of CD31, Ter119 (lymphocyte antigen 76) and CD45, have been established to isolate and study adipocyte progenitor cells. However, these markers are not known to have a functional role in adipocyte development nor do they provide information on the developmental origins of adipocytes [16]. In vivo cellular lineage tracing tools have been developed and implemented to understand the developmental origins of adipocytes. It was observed that embryonic mesenchymal precursor cells expressing Engrailed 1 (En1) give rise to interscapular BAT, dermis and skeletal muscle [16,22]. Furthermore, it was shown that brown preadipocytes from interscapular BAT express myogenic factor 5 (*Myf5*) and myoblast determination protein 1 (*MyoD*). This BAT had a gene expression profile that was similar to that of skeletal muscle rather than preadipocytes of perigonadal WAT [23]. Another lineage tracing study using a Myf5-Cre knock-in allele, showed that interscapular BAT and skeletal muscle are positive for *Myf5* while inguinal and perigonadal WAT are negative for *Myf5* [24]. A similar result was obtained with the use of paired box transcription factor 7 (Pax7)-Cre [25], confirming that BAT and WAT development are from different precursors, with BAT and muscle sharing a common *Myf5+* and *Pax7+* precursor cell, while WAT arises from a different lineage. More recently, it has been shown using *Myf5* labelling to distinguish between BAT and WAT lineages, that interscapular, anterior, and retroperitoneal WAT was labelled with the same Myf5-Cre knock-in allele used in the BAT studies, suggesting that the situation is more complex than previously demonstrated [26,27]. Furthermore, studies combining Myf5-Cre with a dual fluorescent mTmG reporter, used for labelling adipocytes [28], confirmed that unilocular white adipocytes present in the interscapular, anterior, and retroperitoneal WAT depots originated from Myf5-Cre expressing precursors, and that not all brown adipocytes come from Myf5-Cre expressing cells [29]. This study also showed that only half of the adipocytes in the cervical BAT depot were labelled with Myf5-Cre, and none in the perirenal or periaortic BAT were labelled, while all adipocytes in the subscapular and interscapular BAT depots were marked with Myf5-Cre. These observations in BAT and WAT were consistent even when a Pax3-Cre knock-in driver was used. This suggests that a distinct pool of brown and white adipocyte precursor cells exist that arise from embryonic *En1+*, *Pax3+* and *Myf5+* mesenchymal precursors [29].

It is evident from lineage tracing studies that adipocytes arise from multiple lineages that are dynamic and heterogeneously distributed. Also, not all precursor cells that express *Myf5* give rise to BAT and skeletal muscle, since some *Myf5* promoter expressing precursor cells also give rise to white/brite adipocytes. Furthermore, it is not known if brite adipocytes in subcutaneous tissue arise as a result of trans-differentiation or interconversion of pre-existing mature UCP1 negative white adipocytes [30,31], or whether they arise de novo from precursor cells [32]; there is nonetheless strong evidence in support of both models. It is therefore important to understand the developmental origins of adipocytes in vivo to help identify adipocyte precursor cells and the distribution patterns and metabolic differences of the different fat depots, as this could provide opportunities to engineer the development of a particular type of adipocyte (brown or white or beige) for potential health benefits.

## 3. The Adipocyte Formation Process (Adipogenesis)

Adipogenesis is a complex multi-step process that involves the differentiation of MSCs into mature, lipid containing adipocytes [8,33,34]. Two phases have been recognized: commitment and terminal differentiation. Commitment involves the commitment/conversion of MSCs into preadipocytes followed by terminal differentiation into mature adipocytes [35,36]. MSCs become committed to the adipocyte lineage and lose their ability to differentiate into other cell types (osteocytes, chondrocytes, myocytes etc.), while at the same time undergoing morphological and functional changes [36]. The processes of preadipocyte commitment and differentiation involve numerous signalling pathways as well as multiple transcription factors and genes [8,33,34]. Although several signalling pathways have been implicated, this review will focus on those that have been described to play a role in preadipocyte commitment and differentiation, as well as transcription factors involved in regulating adipogenesis. Recent studies have also implicated epigenetics in regulating gene expression during adipogenesis [36]. The epigenetic factors that play a role in adipogenesis such as chromatin remodelling complexes, epigenomic readers, histone methyltransferases/demethylases, histone acetylases/deacetylases, DNA methylases/demethylases, and miRNAs, will also be discussed.

## 4. Regulation of Adipogenesis Via Signalling Pathways

Several signalling pathways have been described to play a role in adipocyte differentiation (summarized in Table 1). 

### 4.1. Insulin-Like Growth Factor 1 (IGF-1) Signalling

Preadipocytes have a large number of IGF-1 receptors relative to insulin receptors. Both IGF-1 and insulin bind to IGF-1 receptors to induce preadipocyte differentiation. However, insulin only binds to the IGF-1 receptor at nonphysiologically high concentrations to mimic IGF-1 activity. Furthermore, stimulation of preadipocyte differentiation by growth hormones occurs through paracrine or autocrine activity by stimulating IGF-1 secretion. IGF-1 is therefore the true inducer of preadipocyte differentiation in vitro. IGF-1 induces differentiation at physiological concentrations that are much lower than insulin and also binds more tightly to the IGF-1 receptor [37]. Zhang et al. (2003) showed that ectopic expression of the full length preadipocyte factor (Pref-1) in 3T3-L1 or 3T3-F442A cells only inhibited differentiation when IGF-1 or insulin were absent from the adipogenic differentiation cocktail [38]. They demonstrated further that the p42/p44 mitogen-activated protein kinase (MAPK) pathway that is compromised in preadipocytes overexpressing Pref-1, was rescued by IGF-1 and insulin to allow for clonal expansion and terminal differentiation. This suggests that IGF-1/insulin bypass the Pref-1 blockade of preadipocyte differentiation [38]. Primary cilium formation occurring at the growth arrest stage during differentiation in confluent 3T3-L1 cells, renders IGF-1 receptors more sensitive to insulin than the IGF-1 receptors not located in cilia. The insulin receptor substrate 1, a downstream molecular target of IGF-1 receptor signalling, is recruited to the basal body during cilium formation and is phosphorylated by receptor kinase in cilia [39]. Another IGF-1 receptor signalling molecule, also activated at the basal body during cilium formation, is Akt-1, also known as protein kinase B (PKB). The inhibition of cilium formation in 3T3-L1 cells by suppressing intraflagellar transport protein 88 homolog (IFT88) or Kinesin family member 3a (Kif3a), blocked IGF-1 receptor signalling, thereby suggesting that the formation of the primary cilium and its basal body during growth arrest induces differentiation in preadipocyte through IGF-1 receptor signalling [39]. Finally, mice with tissue specific double knockout of insulin and IGF-1 showed a significant decrease in both white and brown fat mass, and were resistant to high fat diet-induced obesity and glucose intolerance [40]. These mice showed decreased brown fat activity and were unable to maintain body temperature when kept at 4 °C, but were responsive to β3-receptor stimulation. This suggests that insulin and IGF-1 not only play a role in WAT adipogenesis, but are crucial for brown fat development as defective thermogenesis occurs when they are disrupted [40].

### 4.2. Glucocorticoid (GC) Signalling

Glucocorticoids are steroid hormones that play an essential role in regulating adipogenesis and are included in most adipogenic differentiation media. They transmit a signal through an intracellular glucocorticoid receptor (GR), that subsequently regulates transcription factors [41]. Dexamethasone (Dex), the synthetic GC present in adipogenic differentiation cocktails, is a potent inducer of adipogenesis in vitro [42]. Preadipocytes from humans express GC receptors through which GCs stimulates the expression of PPARγ and C/EBPα during adipogenesis [43,44]. Pref-1 is also reported to be a target for GCs, as Dex has been shown to attenuate Pref-1 expression during adipogenesis in a dose-dependent manner, and therefore could be one of the mechanisms through which GCs promote preadipocyte differentiation [45]. In a more elegant experimental design, 3T3-L1 preadipocytes treated with Dex for 48 hrs followed by a further 48 h of treatment with methylisobutylxanthine (IBMX), induced adipogenic differentiation, while treatment firstly with IBMX followed by Dex did not induce any significant differentiation and had low expression of PPARγ and C/EBPα. This observation was consistent even when C3H10T1/2 were used instead of the 3T3-L1 preadipocytes [42]. It was further observed that Pref-1 expression was inhibited by Dex-to-IBMX treatment and not by IBMX-to-Dex treatment. This suggests that Dex primes preadipocytes into a novel intermediate cellular state during differentiation in vitro, that may be defined by the inhibition of Pref-1 expression [42]. 

### 4.3. cAMP Signalling

Cyclic AMP signalling is primarily mediated through cAMP-responsive element-binding protein (CREB). cAMP, through its cellular target protein kinase A (PKA), phosphorylates and activates CREB, which binds to the Cyclin D1 promoter to activate transcription in the early stages of adipogenesis, thereby promoting 3T3-L1 differentiation [46]. Ectopic expression of cAMP signalling targets CREB in 3T3-L1 cells and stimulates differentiation [47]. The active phosphorylated CREB interacts with the C/EBPβ promoter only after adipogenic induction of 3T3-L1 cells, suggesting that active CREB activates C/EBPβ expression to promote adipogenesis [48,49]. Mouse embryonic fibroblast (MEF) differentiation into adipocytes was markedly inhibited in CREB−/− MEFs [49]. This demonstrates that cAMP, through CREB, activates C/EBPβ expression in the early stages of adipogenesis in 3T3-L1 preadipocytes.

Petersen et al. (2003) showed that an exchange protein directly activated by cAMP (Epac) is required for cAMP dependent activation of adipocyte differentiation [50]. Epac, in synergy with PKA of the cAMP signalling pathway, works via Ras-like GTPases, Ras-related protein 1 (Rap1) and Rap2 (as a guanine nucleotide exchange factor) to promote adipogenesis. The function of PKA in this scenario is to downregulate the activity of Rho and Rho-kinase that suppress the proadipogenic action of IGF-1. This interplay between Epac and PKA demonstrates another mechanism of cAMP signalling that uses both Epac and PKA to drive adipocyte differentiation in 3T3-L1 cells [50].

Both GC and cAMP signalling pathways positively regulate preadipocyte commitment and differentiation [42,50]. However, it is important to note that the different preadipocyte cell lines each provide their own unique perspective in the study of adipogenesis. For example, GCs and cAMP signalling are both required for 3T3-L1 preadipocyte differentiation. This is not the case with Obl771 preadipocytes, in which glucocorticoids alone are sufficient to stimulate high levels of cAMP required for differentiation [51]. 

### 4.4. TGF-β Signalling

Transforming growth factor beta inhibits preadipocyte commitment through mothers against decapentaplegic 3 (SMAD3) signalling, by phosphorylating and suppressing PPARγ expression as well as the expression of C/EBPs [52,53]. Deletion of transforming growth factor beta receptor 2 (*Tgfbr2*) in MSCs resulted in a marked increase in adipocyte expansion in murine bone marrow and this was accompanied by an increase in PPARγ expression [54]. Transforming growth factor beta 1 is the most abundant growth factor in bone matrix and regulates cell growth and differentiation. Human bone marrow mesenchymal stromal/stem cells (BM-MSCs) treated with TGF-β1 for up to 7 days, showed reduced adipogenic differentiation in favour of osteogenic differentiation [55]. Global gene expression analysis revealed that serpin peptidase inhibitor clade B (ovalbumin) member 2 (*SERPINB2*) was significantly downregulated in TGF-β1 treated cells. Silencing of *SERPINB2* in untreated cells enhanced both their adipogenic and osteogenic differentiation capacity. This suggests that TGF-β signalling plays a role in both adipogenic and osteogenic differentiation, and *SERPINB2* was identified as the TGF-β1 responsive gene through which it negatively regulates human BM-MSCs differentiation [55]. Skeletal unloading in rats caused a progressive increase in C/EBPα and C/EBPβ followed by PPARγ2 transcripts in BM-MSCs from day 5 to 7. The administration of TGF-β2 to these rats reversed the effects caused by skeletal unloading. The resultant suppression of PPARγ2 following TGF-β2 administration was associated with higher runt-related transcription factor 2 (Runx2) expression [52]. Furthermore, initial suppression of C/EBPα and C/EBPβ by TGF-β2 increased serine phosphorylation of PPARγ, which inhibited its transactivation activity and suppressed BM-MSCs adipogenic differentiation. Hence, TGF-signalling through TGF-β2 suppressed adipogenesis in BM-MSCs in vivo by inhibiting expressionof C/EBPα, C/EBPβ, and PPARγ [52]. 

### 4.5. BMP Signalling

Bone morphogenetic protein 4 signalling is important in the preadipocyte commitment process, and has been shown to commit C3H10T1/2 pluripotent cells to the adipocyte lineage [56]. BMP4 binds to bone morphogenetic protein receptor type 1A (BMPr1A) and BMPr2 receptors which phosphorylate SMAD1/5/8 to form a complex with SMAD4. This complex is translocated to the nucleus to regulate BMP4 signalling to target genes such as translationally controlled tumour protein 1 (TPT1), lysyl oxidase (LOX) and αB-crystallin, which are involved in the commitment of C3H10T1/2 cells to the adipocyte lineage [56]. BMP4 treated C3H10T1/2 cells differentiated into adipocytes with increased expression of C/EBPα, PPARγ, and adipocyte protein 2 (aP2) [53]. BMP4 pre-treated C3H10T1/2 cells implanted subcutaneously into athymic mice developed into adipose tissue similar to that found in normal fat depots [57]. Treatment of A33 preadipocytes derived from C3H10T1/2 cells with the BMP4 antagonist noggin, inhibited adipogenic differentiation, indicating the importance of BMP4 in maintaining preadipocyte commitment [58]. Inhibiting BMP4 signalling in human adipose derived stromal/stem cells (hASCs) suppresses adipogenesis [53]. Adipose precursor cells secrete Wnt1 inducible signalling pathway protein (WISP2) which forms a complex with zinc finger protein 423 (Zfp423) in the absence of BMP4 stimulation. BMP4 phosphorylates SMAD1/5/8 leading to the dissociation of the WISP2/Zfp423 complex and the release of Zfp423, which in turn activates PPARγ transcription in the nucleus, thereby committing cells to the adipogenic lineage [53].

The role of BMP2 signalling in preadipocyte commitment is not fully understood, but several studies have implicated it in the commitment of C3H10T1/2 cells to this lineage [53,59]. BMP2 committed C3H10T1/2 cells exhibit a certain level of plasticity in differentiation between the different lineages (adipogenesis, chondrogenesis, and osteogenesis), with adipogenesis being particularly favoured at low concentrations of BMP2 [59]. BMP2 induces C3H10T1/2 adipocyte commitment through the activation of SMAD1 and p38 kinase, which stimulate PPARγ expression [60]. Interestingly, the adipogenic effect of BMP2 is greater in hASCs obtained from older than from younger individuals [53].

BMP7 predominantly plays a role in brown adipocyte lineage commitment [53]. This, however, is concentration dependent, with BMP7 at low concentrations promoting adipocyte differentiation in mouse BM-MSCs, while adipogenesis is inhibited at higher concentrations [55]. BMP7 also promotes adipogenic differentiation in human BM-MSCs, but not osteogenic nor chondrogenic differentiation [61]. BMP7 combined with BMP4 induced the expression of UCP1 in hASCs, and could possibly play a role in white to brown (brite) adipocyte formation [62,63]. BMP7-induced adipogenesis led to increase lipid accumulation and PPARγ expression. BMP7 induced brite adipocyte formation by increasing UCP1 expression and decreasing transcription factor 21 (TCF21) (white specific marker) in hASCs, and this was found to be donor dependent [63]. BMP7-treated C3H10T1/2 cells that were implanted subcutaneously into the sternal region of athymic nude mice differentiated to form adipose tissue containing brown adipocytes in vivo [64].

### 4.6. Wnt Signalling Pathway

Wingless-type MMTV integration site family members are glycoproteins that play an essential role in various cellular processes, including embryogenesis, cell proliferation, and cell fate determination [65,66]. There are 19 Wnt genes that encode cysteine-rich glycoproteins that act in an autocrine or paracrine manner [65,67]. The Wnt proteins activate either a Wnt/β-catenin dependent pathway (canonical pathway) or a Wnt/β-catenin independent pathway (non-canonical pathway). Several studies have shown that β-catenin is essential for the regulation of adipogenesis [67,68]. Wnt proteins are secreted into the extracellular environment and activate a cascade of intracellular signals [66]. Wnt proteins bind to a cell surface receptor complex consisting of Frizzled receptor (FZD) and its co-receptor, low density lipoprotein receptor related protein 5/6 (LRP) [65,69]. Once the Wnt protein is bound to the receptor complex, a signal is transduced via phosphoprotein Dishevelled (Dsh), resulting in the inactivation of the β-catenin destruction complex. The β-catenin destruction complex consists of Axin, casein kinase 1α (Ck1α), protein phosphatase 2A (PP2A), adenomatosis polyposis coli (APC) and glycogen synthase kinase 3 (GSK3). Inactivation of this destruction complex prevents the phosphorylation and degradation of cytosolic β-catenin, thereby stabilizing it for translocation to the nucleus [66,70,71]. In the nucleus, β-catenin binds to the T-cell factor/lymphoid enhancer-binding factor (TCF/LEF) family of transcription factors, resulting in the activation of Wnt target genes/transcription factors controlling myogenesis [MYC, cell cycle regulator cyclin D1 (CCND1), and axis inhibition protein 2 (AXIN 2)] [67].

The Wnt signalling pathway has been extensively studied and is a negative regulator of adipogenesis. Several in vitro studies have found that the Wnt signalling pathway inhibits the terminal differentiation of preadipocytes into mature adipocytes [65,72]. Following the activation of the Wnt pathway, the expression of proadipogenic transcription factors (C/EBPα and PPARγ) is inhibited [70]. When 3T3-L1 cells were induced to undergo adipogenic differentiation, there was an increase in the expression of PPARγ, C/EBPα and adducin 1 (Add1), as well as the adipogenic genes, aP2 and adiponectin (APM1). However, in a model in which the Wnt signalling pathway was activated, there was little to no expression of PPARγ, C/EBPα, Add1, aP2 and APM1 [69]. Thus, inhibition of this pathway results in the formation of mature adipocytes [65,69].

### 4.7. Hedgehog Signalling Pathway

The Hedgehog (Hh) signalling pathway plays a role in embryogenesis and cell differentiation [73]. Activation of the Hh signalling pathway inhibits adipogenesis and promotes osteogenesis and chondrogenesis [74]. This signalling pathway involves the binding of extracellular Hh protein to a cell surface receptor complex consisting of Patched (Ptch) and Smoothened (Smo). Smo then activates a cascade of intracellular signals resulting in the activation of target genes by Gli family transcription factors (Gli 1, 2, and 3) [75]. Adipocyte differentiation is prevented through the specific inhibition of C/EBPα and PPARγ [35]. A study by Suh et al. (2006) using 3T3-L1 cells showed that activation of the Hh pathway inhibited adipocyte differentiation; cells retained the appearance of uninduced 3T3-L1 cells and there was a reduction in lipid accumulation. The authors further observed reduced expression of C/EBPα, PPARγ, aP2, and Adiposin, while Pref-1 levels were elevated [76].

### 4.8. MAPK Signalling Pathways

The intracellular MAPK signalling pathway is important for cell proliferation and differentiation. It is subdivided into three pathways: extracellular signal-regulated kinases (ERKs), c-Jun amino-terminal kinases (JNKs) and p38 MAPK. The ERK and p38 MAPK pathways have been implicated in the regulation of adipogenesis [77]. Extracellular signal-regulated kinase signalling plays a role during the early stages of adipogenesis, as ERK1-/- mice were protected against high fat diet induced obesity with a decrease in adiposity. Preadipocytes from these mice as well as embryo fibroblasts showed impaired adipogenesis [78]. Contrary to the reports on the involvement of ERK in early adipogenesis, other studies have shown that sustained activation of ERK decreases adipogenesis by inhibiting PPARγ expression through MAPK mediated phosphorylation [79,80]. There have been contradictory reports on the role of the p38MAPK pathway in adipogenesis [77,81,82,83]. In p38MAPKalpha knockout cells or cells in which p38MAPK has either been inhibited or disrupted, phosphorylation of CEBPβ was enhanced and PPARγ expression increased, suggesting that p38MAPK plays a negative role in adipogenesis [77]. Conversely, an increase in p38MAPK activity has been observed during human preadipocyte differentiation, and using pharmacological substances to inhibit p38MAPK in these cells greatly reduces the accumulation of triglycerides and the expression of PPARγ together with other adipocyte specific markers. This suggests that p38MAPK plays a positive role in primary human preadipocyte differentiation [81]. Another study showed that specific inhibitors of p38 blocked adipogenesis in 3T3-L1 cells. Treatment with a p38 inhibitor reduced CEBPβ phosphorylation in vivo with a corresponding decrease in PPARγ expression. This suggests that CEBPβ may be a target for p38 during adipogenesis, and that p38 MAPK activity promotes 3T3-L1 differentiation during the initial stages of adipogenesis [82,83].

### 4.9. Other Signalling Pathways

The Ras signalling pathway induces 3T3-L1 preadipocyte differentiation; ectopic expression of the Ras oncogene induces preadipocyte differentiation in the absence of insulin and IGF-1 [37]. Further evidence suggests that activated Ras mediates its adipogenic effect through cytosolic serine/threonine protein kinase rapidly accelerated fibrosarcoma 1 (Raf-1), as the expression of Raf-1 was sufficient to induce preadipocyte differentiation [37,84]. Retinoblastoma protein (pRb) in the Rb signalling pathway binds to E2 transcription factor (E2F) to repress its activity, thereby inhibiting cell-cycle progression. pRb also acts with E2F to inhibit the expression of the PPARγ2-c subunit and subsequently adipogenesis [85]. Cyclin-dependent kinase phosphorylates pRb which can either suppress or promote adipogenesis, depending on the cellular context and activity of the transcription factor induced. pRb releases E2F which activates cell-cycle genes required for synthesis phase (S-phase) entry and cell-cycle progression, that are critical for mitotic clonal expansion [48,86]. Conversely, pRb can also bind to RUNX2, inhibiting adipogenesis in favour of osteogenic differentiation [87]. Myostatin signalling inhibits 3T3-L1 preadipocyte differentiation and BMP7-induced C3H 10T1/2 adipogenic differentiation in vitro [88] (Table 1).

## 5. Epigenetic Regulation of Adipogenesis

Epigenetic mechanisms play a crucial role in regulating gene expression and chromatin structure and result in heritable changes in gene expression without altering DNA sequences. A broadened definition of epigenetics includes any alteration of chromatin or DNA that effects gene expression, and this includes post translational modifications to proteins such as acetylation, methylation and phosphorylation, histone modifications, RNA silencing, non-coding RNA regulation including miRNAs, short interfering RNAs (siRNAs), piwi-interacting RNAs (piRNAs) and long non-coding RNAs (lncRNAs), protein phosphorylation, genomic imprinting, cell differentiation, and environmental factors. This review will focus on those epigenetic modifiers acting at the genomic level with a well described role in adipogenesis, such as chromatin remodelling complexes, epigenomic readers, histone methyltransferases/demethylases, histone acetylases/deacetylases, DNA methylases/demethylases, and miRNAs (Table 2 and Table 3).

### 5.1. Chromatin Remodelling Complexes

Remodelling of chromatin architecture renders DNA accessible during replication, transcription, and DNA repair, thereby allowing for changes in gene expression. Remodelling occurs through covalently modified histones and ATP-dependent chromatin remodelling factors [89]. The switch/sucrose nonfermenting family is a multi-subunit ATP-dependent chromatin remodelling complex that makes use of mammalian brahma (BRM) or brahma-related bromodomain protein (BRG1) as ATPase [90]. Salma and colleagues (2004) analysed interactions between PPARγ2 and SWI/SNF. They found that prior to the interaction of SWI/SNF enzymes with the PPARγ2 promotor, changes occurred in the binding of C/EBP activators, general transcription factors, and polymerase II. In contrast, PPARγ2 transcription only occurred after the association of SWI/SNF with transcription factor II H (TFIIH). Therefore, adipogenesis proceeds when SWI/SNF is recruited to PPARγ2 [90,91].

### 5.2. Histone Methylation

Histones are a family of alkaline proteins and are of five major types: H1, H2A, H2B, H3, and H4. Their main function is to package DNA to form structural units called nucleosomes [92]. Histone methylation allows for either the activation or repression of transcription and is dependent on the methylation of lysine or arginine residues [93]. Little is known about histone marks in human adipose tissue; however, it has been shown that alterations in histones are an essential element in the epigenetic regulation of adipogenesis. Examples of the effect of histone methylation on gene expression include methylation of histone 3 lysine 4 (H3K4) that can be mono-, di-, and tri-methylated, as well as H3K36 and H3K79, all of which are associated with gene activation. On the other hand, methylation of H3K9 and H3K27 is related to gene repression [94]. C/EBPβ and H4 promotors are involved in cell progression through growth phase 1 (G1) as a result of C/EBPβ binding to and transactivation of H4 promoters, thereby increasing H4 expression. Musri and colleagues (2006) observed H3K4 di-methylation at the APM1 promoter in 3T3-L1 fibroblasts, indicating transcriptional competence, which represents a marker for cells that have undergone determination to the preadipocyte stage. The authors knocked down the H3K9 methyltransferase, SET domain bifurcated histone lysine methyltransferase 1 (SETDB1); this decreased H3K9 and increased H3K4 di-methylation at the C/EBPα promoter, resulting in an increase in C/EBPα gene expression [95,96]. 

Lysine specific demethylase 1 (LSD1) plays a role in both the activation and repression of transcription, depending on the biological processes and associated protein complex [90]. A study by Musri and colleagues (2010) demonstrated the effect of LSD1 on the differentiation of 3T3-L1 preadipocytes. Knockdown of the H3K4/K9 demethylase LSD1 decreased 3T3-L1 cell adipogenic differentiation. This resulted in an increase in H3K9 di-methylation and a decrease in H3K4 di-methylation at the promoter region of C/EBPα, which impaired C/EBPα activation. Hence, knockdown of LSD1 increases the levels of histone repressive markers, which inhibits adipogenesis [95]. LSD1 is essential for the development and function of BAT. Sambeat and colleagues (2016) assessed *Myf5*+ progenitor cells in which LSD1 had been knocked out, and found an increase in H3K9 di-methylation (H3K9me2) on the UCP1 promoter. This inhibited UCP1 expression, resulting in defective BAT function and development. The authors then undertook a study in UCP1-Cre mediated LSD1 knockout mice, and found an increase in adipose tissue as well as cold intolerance [97]. During adipogenesis, G9a and H3K9me2 levels decreased to enhance chromatin opening and binding of C/EBPβ to the PPARγ promoter, thereby activating the expression of PPARγ. In addition, G9a knockout mice showed an increase in both WAT and BAT [98].

Nuclear receptor binding SET domain protein 2 (Nsd2) is an H3K36 di-methyltransferase (H3K36me2) and is involved in the positive regulation of adipogenesis. The depletion of Nsd2 has no effect on H3K36me2 but increases H3K27 tri-methyltransferase (H3K27me3), which in turn represses adipogenesis [99]. In vivo studies showed that LSL-H3.3K36M;Myf5-Cre mice had a decrease in BAT and an abnormal hunched posture [99]. Fatty acid binding protein 4 (FABP4) promoter driven H3K36M transgenic mice exhibited whitening of BAT and developed insulin resistance in WAT. This shows that Nsd2 is essential for the development and function of adipose tissue [99]. Lysine demethylase 5 (Kdm5), a family of H3K4 demethylases, is involved in cell cycle progression where it binds to and activates specific promoters. Knockdown of Kdm5 results in inhibition of adipogenesis as a result of reduced mitotic clonal expansion [100]. However, the role of Kdm5 in vivo is not yet fully understood. 

Zhang and colleagues (2012) stated that “global levels of histone modifications remain stable during adipogenesis” [101]. Using 3T3-L1 cells, they observed an increase in H4K20 mono-methylation from day 0 to day 2. During clonal expansion, at time points of 6 hrs and 1 day, there was a general loss of histone 3. Using chromatin immunoprecipitation (ChIP) analysis, they examined the different types of methylation on H3K4. They found that the C/EBPβ exon and 3′UTR had only a small amount of K4 mono-methylation but contain a large amount of K4 tri-methylation. In addition, high levels of K4 tri-methylation were present in all the induced adipogenic genes. From these findings they concluded that there is a positive correlation between H3K4 tri-methylation and gene activation in adipogenesis [101]. A study by Ge (2012) showed that H3K4 methyltransferase MLL3/4 and H3K27 demethylase tetratricopeptide repeat protein (UTX; also known as KDM6A) control the expression of PPARγ and C/EBPα. Both histone modifiers associate with paired box transcription activation domain interacting protein (PTIP) [102]. MLL3/4 are required for the activation of adipogenesis through the induction of PPARγ and C/EBPα. During adipogenesis, MLL3/4 are recruited to further activate downstream enhancers. However, in in vivo studies in Myf5-Cre-mediated MLL4 knockout mice, these mice had defects in BAT, and adipogenesis was inhibited [103]. Wnt genes are repressed by H3K27 methyltransferase (PRC2) and its subunit Ezh2, which in turn promotes adipogenesis. These results indicate that methylation facilitated by H3K4 and H3K27 controls the expression of the main genes involved in adipogenesis [94,102]. In the regulation of BAT, Ehmt1 is essential for stabilizing the PR domain containing 16 (PRDM16) protein that is required for thermogenesis. Ohno et al. (2013) produced Myf5-Cre-mediated Ehmt1 knockout mice that showed a reduction in BAT-mediated adaptive thermogenesis, systemic insulin resistance, as well as obesity. This suggests that Ehmt1 plays an essential role in controlling cell fate in BAT and energy homeostasis [104]. 

In addition to lysine methylation, arginine methylation is also implicated in adipogenesis. In mammals, 9 PRMTs mediate arginine methylation, of which PRMT5 and CARM1 play a role. CARM1 is recruited to promoters and is a transcriptional coactivator for PPARγ, hence promoting adipogenesis [90]. Yadav and colleagues (2008) knocked down CARM1 in 3T3-L1 cells which inhibited adipogenesis, thus indicating that it is an important element in the activation of PPARγ. CARM1 knockout embryos displayed decreased lipids in BAT, indicating that CARM1 is necessary for differentiation into mature adipocytes [90,105]. PRMT5 is required for enhancer promoter loop formation of PPARγ2 and demethylates histones at adipogenic promoters. LeBlanc and colleagues (2012) found that the presence of PRMT5 promotes the binding of ATP-dependent chromatin remodelling enzymes that are essential for PPARγ2 binding to PPARγ2-regulated promoters. When PRMT5 is overexpressed, adipogenesis increases; on the other hand, the inhibition of PRMT5 resulted in the repression of adipogenic genes. Thus, PRMT5 plays a role in coactivation for adipogenic gene expression and adipogenesis [106,107]. 

### 5.3. Histone Acetylation

Histone acetylation plays an important role in gene expression. It involves the addition of acetyl groups on the histone N-terminal tail by histone acetyl transferases (HAT). There are two types of HAT: Type A contains a bromodomain, that is found in the nucleus and acetylates chromatin and nucleosomal histones, for example Gcn5/PCAF and CBP/p300; Type B is found in the cytoplasm and acetylates newly transcribed histones [108]. Acetylation results in a relaxed chromatin structure that allows for transcriptional activation [109]. Histone tails H3 and H4 contain many acetylated sites and therefore are involved in positive gene expression. General control non-depressible 5 and PCAF are involved in the acetylation of H3K9 and play a role in adipogenesis by regulating the expression of PPARγ and PRDM16. Jin and colleagues (2014) demonstrated that a double knockout of Gcn5 and PCAF prevented adipocyte differentiation and BAT development by inhibiting PPARγ expression. Ectopic expression of PPARγ was able to rescue the adipogenic defects caused by the double knockout, but not brown adipocyte enriched PRDM16 expression. These results indicate that WAT adipogenesis is regulated by Gcn5 and PCAF through PPARγ expression, and brown adipogenesis is regulated by influencing the expression of PRDM16. Hence, the transcription of general white adipogenic genes and brown adipogenesis is regulated through different mechanisms [110]. A study using 3T3-L1 cells showed that ribozyme-mediated targeting of CBP or p300 inhibited the expression of PPARγ. This indicates that CBP and p300 are required for the induction of PPARγ [111]. CBP deficient mice showed a decrease in WAT but not in any other tissues. These mice displayed an increase in insulin sensitivity and glucose tolerance [112]. Even though CBP and p300 contain similar sequences, they appear to be involved at different time points during adipogenesis [111].

Using ChiP analysis and 3T3-L1 cells, Zhang et al. (2012) analysed H3K9/K14 and H4K12 acetylation in relation to adipogenesis. Like H3K4 tri-methylation, C/EBPβ exon and 3′UTR were highly acetylated during H3K9/K14 acetylation, but not in H4K12 acetylation. In addition, the aP2 gene showed the highest amount of histone acetylation. PPARγ2 and aP2 showed increased levels of acetylation on both H3 and H4 tails [101]. Xu, Ande, and Mishra (2013) examined temporal changes in acetylation of protein lysine in 3T3-L1 cells during adipogenesis over an eight-day period. The cells were analysed using SDS-PAGE (sodium dodecyl sulphate-polyacrylamide gel electrophoresis) and immunoblotting with anti-lysine acetylation specific antibody [113]. During days 1–4 of adipogenesis, downregulation of protein acetylation occurred, while during days 4–8 upregulation took place. Since histone deacetylase (HDAC) inhibitors play a role in the early stages of adipogenesis, these results demonstrate that downregulation of protein acetylation is critical for adipocyte differentiation. 

Bromodomain-containing protein 4 is a member of the bromodomain and extraterminal domain (BET) protein family, and acts as an epigenetic reader by binding to acetylated histones and transcription factors that promote the expression of PPARγ. The disruption of BRD4 in BRD4 knockout cell lines inhibits PPARγ expression and suppresses adipogenesis [114]. Lee and colleagues (2017) demonstrated that BRD4 binds to active enhancers through enhancer epigenetic writers MLL3/4 during adipogenesis, which facilitates the recruitment of positive transcription elongation factor (p-TEFb), RNA polymerase II (Pol II) and transcription factor II D (TFIID) [90,115]. BRD4 knockout models showed a decrease in BAT and muscle mass in vivo, and the mice displayed an abnormal hunched posture. This indicates that BRD4 is an important factor for adipogenesis as well as myogenesis in vivo [115].

### 5.4. Histone Deacetylation

Histone deacetylases (HDACs) are involved in chromatin modification. HDACs deacetylate histones (removal of an acetyl group), compacting the chromatin structure and preventing the binding of transcription factors. There are 4 classes of HDACs. Class I consists of HDACs 1, 2, 3, and 8. Class II consists of HDACs 4, 5, 6, 7, 9 and 10. Class III consists of sirtuins 1–7, a group of nicotinamide adenine dinucleotide (NAD)-dependent enzymes. Class IV consists of HDAC 11 [116,117].

HDAC 1 negatively regulates adipogenesis. Eung and colleagues (2006) observed that levels of HDAC 1 expression were reduced during adipogenesis. Further investigation identified HDAC 1 as a possible regulator of PPARγ, C/EBPα, sterol regulatory element binding transcription factor 1c (SREBP-1c) and aP2 expression [117]. Although this study identified a role for HDAC 1 in regulating adipogenesis, controversy still remains regarding its role [118]. Haberland and colleagues (2010) found that HDACs 1 and 2 together promote adipogenesis, as their deletion inhibited this process [119]. As the role of HDAC 1 is not completely understood, further research is required. HDAC 9 negatively regulates adipogenesis. Chatterjee and colleagues (2011) found that HDAC 9 inhibited adipogenesis in 3T3-L1 cells. It was subsequently found that HDAC 9 forms a complex with upstream transcription factor 1 (USF-1) and interacts with the promoter of the C/EBPα gene. Knockout of HDAC 9 resulted in increased expression of C/EBPα in vivo, possibly indicating the target of HDAC 9 [120].

Histone deacetylases class III consists of 7 Sirt enzymes that regulate various cellular processes by deacetylating lysine residues. A few of the Sirt enzymes have been implicated in adipogenesis [121,122]. Sirt 1 is a negative regulator of adipogenesis. Picard and colleagues (2010) observed that a reduction in Sirt 1 resulted in increased expression of PPARγ, C/EBPα and aP2. Knockout of Sirt 1 promoted adipogenesis in vivo as well as a decrease in free fatty acid release from WAT [123,124]. Sirt 2 has also been implicated as a negative regulator of adipogenesis. In vitro knockdown of Sirt 2 promoted adipogenesis, and increased expression of PPARγ, glucose transporter type 4 (Glut4), and adipsin. It was also shown that Sirt 2 deacetylates FOXO1, and as a result FOXO1 binds to PPARγ and represses its activity [125]. Sirt 6 is a positive regulator of adipogenesis, playing a role in clonal expansion. In vitro studies using 3T3-L1 cells revealed that knockout of Sirt 6 impaired differentiation. This was confirmed in in vivo studies whereby a reduction in subcutaneous adipocytes was observed and a decrease in expression of PPARγ, C/EBPα, aP2, and APM1 was found. Sirt 6 was found to particularly target kinesin family member 5C (KIF5C), inhibiting its expression during adipogenesis [126]. An antagonistic effect has been reported between Sirt 7 and Sirt 1. Sirt 7 interacts with Sirt 1 leading to Sirt 1 being acetylated. This decreases Sirt 1 activity and as a result, adipogenesis is promoted. Sirt 1 expression is increased in Sirt 7-/- mice. A reduction in WAT was observed as well as a decrease in expression of PPARγ and aP2. Sirt 7 therefore plays a positive role in regulating adipogenesis by inhibiting Sirt 1 [121]. 

### 5.5. DNA Methylation

DNA methylation is a key element in the regulation of gene expression and cell differentiation. It involves the transfer of a methyl group onto the fifth carbon (C5) of cytosine, forming 5-methylcytosine (5mCs). This is facilitated by Dnmts [127,128]. Dnmts have been shown to regulate adipogenesis. Dnmt1 is involved in clonal expansion and the early stages of adipogenesis. High levels of Dnmt1 expression are observed in the first 24 hrs following adipogenic induction, with a subsequent reduction in expression [129]. To further examine the functional role of Dnmt 1 during adipogenesis, PPARγ and Glut4 loci were analysed. Both loci showed increased methylation of 5′-carbon-phosphate-guanine-3′ (CpG5) islands, particularly during the early stages of differentiation, with a decrease in the level of methylation thereafter. Based on these findings, it is believed that Dnmt1 suppresses the expression of adipogenic genes and in turn allows clonal expansion to occur. When Dnmt 1 was silenced, spontaneous differentiation of preadipocytes occurred [129]. Therefore, Dnmt1 appears to promote the early stages of differentiation (clonal expansion) through the methylation of PPARγ, thereby inhibiting its early expression. The level of methylation decreases during the later stages of differentiation, allowing the expression of PPARγ and thereby promoting differentiation.

### 5.6. DNA Demethylation

Ten eleven translocation enzymes are a group of enzymes implicated in the reversal of DNA methylation through the oxidation of 5mCs [130]. Yoo and colleagues (2017) reported that Tet 1 and 2 positively regulate adipogenesis, specifically by reducing DNA methylation as well as inducing hydroxymethylcytosine at the PPARγ locus. In vitro knockdown of Tet 1 and 2 prevented expression of PPARγ and subsequently blocked adipogenesis. It was also found that Tet 2 was mainly responsible for modulating expression at the PPARγ locus [131] (Table 2).

### 5.7. miRNAs

MicroRNAs are small non-coding 20–22 nucleotide RNA sequences, that are critical posttranscriptional regulators modulating the expression of transcription factors and genes in various cellular processes [133]. Several studies have implicated miRNAs in the regulation of adipogenesis (Table 3). Different miRNAs are expressed at various stages of adipogenesis and regulate this process either positively or negatively [134]. 

Esau and colleagues (2004) investigated miRNA expression in preadipocytes and mature adipocytes. Levels of miR-143 expression were found to increase in human white preadipocytes induced to undergo adipogenic differentiation [135], and the inhibition of miR-143 was shown to prevent adipocyte differentiation. MiR-143 targets ERK5, which is an essential component of the MAPK signalling pathway [135,136]. The MiR 17-92 cluster plays a positive role in the clonal expansion phase of adipogenesis. The MiR 17-92 cluster is a highly conserved cluster consisting of miR-17, miR-18a, miR-19a, miR-9b, miR-20a, and miR-92A [137,138]. The miR 17-92 cluster targets retinoblastoma-like protein 2 (RB2/P130) of the retinoblastoma tumour suppressor gene family, also referred to as checkpoint proteins [138]. Ouyang and colleagues (2015) investigated the role of miR-125b-5p in adipogenesis using 3T3-L1 cells, and found that it inhibits cell proliferation while promoting adipogenic differentiation. This was evident from the increase in lipid droplets and the expression of PPARγ, C/EBPα and FABP4. miRNA-125b-5p was found to suppress the G1/S transition as well as to inhibit the expression of G1/S associated genes [139].

The miR-30 family (miR-30 a–d) has been identified as being pro-adipogenic. A study by Zaragosi et al. (2011) showed miR-30 a–d was upregulated in mature adipocytes. It was further reported that miR-30 a and d specifically target RUNX2, a major pro-osteogenic transcription factor, thereby inhibiting osteogenesis and stimulating adipogenesis [140]. Another miRNA that targets RUNX2 and has the same effect on adipogenesis is miR-204 and its homolog miR-211 [141]. MiR-124 also inhibits osteogenesis, but instead of targeting RUNX2, it targets distal-less homeobox 5 (Dlx5), another pro-osteogenic transcription factor [136]. Thus, miR-30 a and d, miR-204, and miR-124 appear to play a role in adipocyte commitment. There have also been several miRNAs reported to suppress or inhibit adipogenesis [69,142]. Expression of MiR-130 results in the inhibition of adipogenesis by specifically targeting PPARγ [142]. Another negative regulator of adipogenesis is miR-27a, which has been shown to target PPARγ and C/EBPα [143]. MiR-210 was found to promote adipogenesis by suppressing the Wnt signalling pathway [69], while MiR-146 promotes adipogenesis by suppressing Sirt 1 and subsequently acetylating FOXO1 [144]. MiR-93 is another miRNA found to inhibit Sirt-7 as well as T-box 3 (Tbx3), and in turn negatively regulates adipogenesis [145] (Table 3).

## 6. Transcriptional Regulation of Adipogenesis

**Figure 2:**
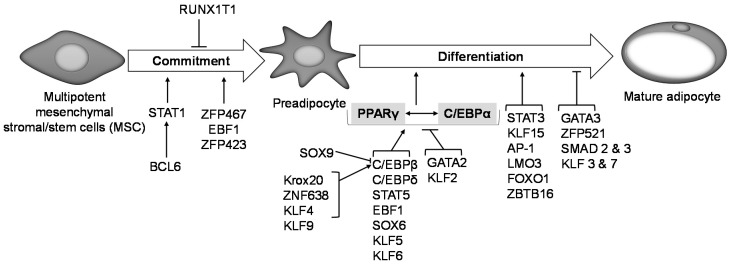
Transcriptional regulation of adipogenesis. Transcription factors Zfp423, Zfp467, EBF1 and BCL6 promote preadipocyte commitment into the adipogenic lineage, while RUNX1T1 inhibits this process. Expression of PPARγ and C/EBPα is central to preadipocyte commitment and terminal differentiation, and several other transcription factors are known to regulate PPARγ and C/EBPα expression downstream. Activation of either PPARγ or C/EBPα transactivates the other. Krox20, ZNF638, KLF 4 and 9 activate C/EBPβ expression, which in turn activates PPARγ and thus promotes adipogenesis. SOX9 on the other hand binds to and suppresses C/EBPβ promoter activity and inhibits adipogenesis. KLF 5, 6 and 9, SOX6, EBF1, STAT5, C/EBPδ activate PPARγ expression thereby promoting adipogenesis. GATA2 and KLF2 inhibit PPARγ activation and suppress adipogenesis. STAT3, KLF15, AP-1, LMO3, FOXO1 and ZBTB16 are other transcription factors are reported to promote preadipocyte differentiation, while GATA3, ZFP521, SMAD 2 and 3, KLF 3 and 7 are reported to suppress it.

The process of adipogenesis has been extensively studied and decades of research have reported over a dozen transcription factors to be involved in regulating this process, both in vitro and in vivo [146,147] (Figure 2). The transcription factor Zfp423 plays an essential role in regulating MSC commitment to preadipocytes and its expression remains unchanged during adipogenesis [148,149]. Ectopic expression of Zfp423 in non-adipogenic NIH-3T3 fibroblasts activates PPARγ expression in undifferentiated cells, which allows them to undergo adipogenesis, hence suggesting that Zfp423 is an important transcriptional regulator of preadipocyte determination [149]. Zinc finger protein B-cell lymphoma 6 (Bcl6) promotes preadipocyte commitment and differentiation in vitro and ex vivo, such that Bcl6 knockdown in C3H10T1/2 cells suppresses their adipogenic potential while its overexpression enhances adipogenesis by activating STAT1 downstream. Impaired adipogenic commitment and differentiation of Bcl6 knockdown C3H10T1/2 cells was rescued by STAT1 overexpression, making STAT1 a direct downstream target of Bcl6 [150]. Intra-tibial injection of cells transduced with Zfp467 into C57Bl/6 mice doubles the number of adipocytes found in the bone marrow compared to vector control-transduced cells, suggesting a potential role for Zfp467 in the commitment of precursor cells to the adipogenic lineage [151]. Early B-cell factor 1 plays a role in adipogenic lineage commitment in mice as shown by the lack of adipocyte precursor cells in Ebf1 null mice [152]. Overexpression of Ebf1 in fibroblasts promotes adipogenic differentiation through the direct activation of the PPARγ promoter [147,153]. STAT5 promotes adipogenesis in vitro and in vivo in murine and human preadipocyte and non-precursor cells [147,154,155,156,157]. Studies in both 3T3-L1 cells and C3H10T1/2 cells show that STAT5 promotes adipogenesis by inducing PPARγ expression [158]. Nude mice injected with a vector expressing STAT5A developed adipose tissue at the site of injection after 6 weeks, while mice receiving control vector failed to develop adipose tissue, suggesting STAT5A promotes adipocyte development [159]. RUNX1 inhibits C3H10T1/2 cell commitment to preadipocytes and its expression in BMP4-treated cells was found to be very low. Forced expression of RUNX1 in BMP4-treated C3H10T1/2 cells inhibited their commitment to the adipocyte lineage, which suggests that downregulation of RUNX1 is needed for C3H10T1/2 adipocyte commitment [160].

A large body of evidence indicates that PPARγ and C/EBPα are the master regulators of adipocyte development and adipogenesis. PPARγ and C/EBPα knockout in murine adipocytes during embryonic and adult development showed that PPARγ was important for adipocyte development in both embryonic and adult stages, while C/EBPα was crucial for adult murine WAT adipogenesis but not in embryonic WAT adipogenesis [161]. The expression of functional PPARγ is an absolute requirement for adipogenesis both in vitro and in vivo [162,163]. C/EBPβ, C/EBPδ, and Kruppel-like factors 5, 6 and 9 are implicated in adipogenesis, and have all been shown to induce PPARγ expression in vitro, while GATA binding protein 2 (GATA2) and KLF2 inhibit PPARγ expression [147,164,165]. Expression of C/EBPβ and C/EBPδ occurs early during adipocyte differentiation to trans-activate C/EBPα and PPARγ [147,166,167]. Krox20 and ZNF638 activate C/EBPβ, thereby modulating adipocyte differentiation. Krox20 stimulates adipogenesis in vitro either through a C/EBPα dependent or independent mechanism [168], while stimulation of adipogenesis by ZNF638 only occurs through the C/EBPs [169].

The expression of STAT 1, 3 5A, and 5B has been shown to increase during adipogenic differentiation in 3T3-L1 cells [147,154]; conversely, in human preadipocytes, STAT1 expression decreases during adipogenesis [154]. It is not clear why the expression of STAT1 differs between murine and human preadipocytes with respect to its role in adipogenesis; however, it is unlikely that STAT1 plays a critical role in adipogenesis in vivo, as STAT1 knockout mice do not show abnormalities in body weight or adiposity phenotypes [170]. KLF 4, and 15 promote adipogenesis, while KLF 3 and 7 inhibit it [147]. KLF4 is expressed very early in adipogenesis and induces C/EBPβ expression, while knockdown of KLF4 inhibits adipogenesis [171]. KLF5 is also expressed early in adipogenesis, and KLF5^+/−^ mice show a significant reduction in WAT [172]. KLF15 was shown to be highly expressed only in mature 3T3-L1 adipocytes [173]; however, we have observed that KLF15 is constantly expressed throughout adipogenesis in vitro using a human preadipocyte model [174]. Nonetheless, both studies suggest a proadipogenic role for KLF15. Overexpression of both KLF 2 and 3 inhibit adipogenesis, with KLF2 directly inhibiting PPARγ2 promoter activity while KLF3 attenuates C/EBPα promoter activity [175,176]. The zinc finger E-box binding homeobox 1 (ZEB1) transcription factor promotes adipocyte differentiation both in vitro and in vivo [177]. Overexpression of another transcription factor, SREBP-1c, in non-precursor cells, enables them to undergo adipogenesis, suggesting a role in adipogenesis in vitro [178]. In vivo studies have revealed a less compelling role for SREBP-1c in adipogenesis, as SREBP-1 knockout mice showed no change in adipose tissue development or the expression of key adipogenic genes when compared to wild-type mice [179]. This may indicate that SREBP-1c expression is not critical for adipogenesis and adipose tissue development. WAT preadipocytes express GATA 2 and 3, and their expression is suppressed during adipogenic differentiation. Enhanced adipogenic differentiation is seen in embryonic stem cells lacking GATA2, while ectopic expression of GATA2 suppresses adipogenesis through direct binding to and deactivation of PPARγ promoter [180]. Sex Determining Region Y-Box 6 and SOX9 have opposite effects on adipogenesis: while SOX6 is proadipogenic, SOX9 inhibits adipogenesis [147]. SOX6 mediates its proadipogenic effect by activating PPARγ and C/EBPα as well as through the inhibition of Wnt/β-catenin signalling [181]. Conversely, SOX9 binds to the promoters of C/EBPβ and C/EBPδ to suppress their activity, thereby inhibiting adipogenic differentiation. Overexpression of SOX9 in cells suppressed while its knockdown increased adipogenesis [182]. The transcription factor activator protein 1 (AP-1) promotes adipogenesis by inducing FABP4 promoter activity [183]. Other transcription factors such as LIM only domain protein 3 (LMO3), FOXO1 and zinc finger and BTB domain containing 16 (ZBTB16), have been shown to be highly expressed during human adipogenesis in vitro, suggesting a possible role in promoting this process [174]. In silico analysis of genes that were differentially expressed during human adipogenesis in vitro [174] has identified several other transcription factors with possible roles in adipocyte differentiation [146]. 

It is evident that many studies over the past few decades have reported many new transcription factors with roles in adipogenesis in vitro; however, for some, their functions in vivo remain to be fully investigated. For those that have been shown to play a role in adipocyte formation in vivo, their clinical translation has been very limited. Hence, more studies are needed to carefully understand the mechanistic role of each transcription factor in order to advance our knowledge on how best to interfere with adipocyte formation for potential health benefits.

## 7. Conclusions

Adipogenesis in WAT is a complex molecular process in which the expression of PPARγ and C/EBPα is key to the formation of a mature white adipocyte. Several molecular factors including signalling pathways, epigenetic modifiers and other transcription factors regulate the expression of these two transcription factors during adipogenesis (Figure 1), which together, regulate the expression of key adipogenic genes that characterize the adipocyte phenotype. PPARγ and C/EBPα expression is inhibited by the canonical Wnt/β-catenin, hedgehog, TGF-β1 and 2, and Rb signalling pathways, while GC, cAMP and BMPs signalling stimulate PPARγ and C/EBPα expression. PRC2 and Ezh2 as well as MiR-210 suppress Wnt genes of the β-catenin pathway thereby promoting PPARγ and C/EBPα expression. BRD4 binds to MLL3/4 to activate PPARγ and C/EBPα transcription. Gcn5 and PCAF acetylate H3K9 while CBP/p300 acetylates H3K27 and H3K18 on the PPARγ promoter to activate transcription. CARM1 acts as a coactivator for PPARγ transcription while SWI/SNF is recruited to PPARγ to activate transcription. Sirt 2 deacetylates FOXO1 causing it to bind to and suppress PPARγ promoter activity. Dnmt1, on the other hand, methylates the CpG5 island on the PPARγ promoter to suppress its expression and allow cells to undergo clonal expansion during the early phase of adipogenesis. Tet 2 demethylates the PPARγ promoter allowing for transcription. miR-130 directly targets PPARγ to suppress its expression, while miR-125b-5p indirectly favours PPARγ expression. PRMT5 promotes PPARγ binding to promoters of its downstream target genes. C/EBPβ is an activator of PPARγ expression. The expression of G9a and H3K9me2 prevent chromatin opening of C/EBPβ, thereby preventing PPARγ transcription. H3K4me3 as well as the acetylation of H3K9/K14 on the C/EBPβ promoter activate transcription. Krox20, ZNF638, KLF4, and KLF9 are transcription factors that promote C/EBPβ expression while SOX9 inhibits it. C/EBPδ, STAT5, EBF1, SOX6, KLF5, KLF6, and KLF9 are transcription factors that promote PPARγ expression, while GATA2 and KLF2 inhibit PPARγ expression. Sirt1 decreases PPARγ and C/EBPα expression while miR-27a directly targets PPARγ and C/EBPα to suppress their expression. On the other hand, MiR-146 and Sirt 7 suppress Sirt1 activity, thereby promoting adipogenesis by allowing for the expression of PPARγ and C/EBPα. MiR-93 directly inhibits the activity of Sirt7 and negatively affects PPARγ and C/EBPα expression. HDAC 9 decreases C/EBPα expression while H3K4me2 increases transcription at the C/EBPα promoter. LSD1 can either increase H3K4me2 or decrease H3K9me2 at the C/EBPα promoter to promote or suppress transcription, respectively. 

To date, PPARγ is the only modulator of adipogenesis that has shown clinical relevance in addressing an obesity associated comorbid condition like diabetes [184,185]. This demonstrates that our understanding of the complex multistep process of WAT adipogenesis and its key modulators is still limited. Increased understanding of the function of the key determinants in adipogenesis and how they relate to adipose tissue functioning, will provide knowledge on how to target them for anti-obesity drug development without compromising metabolic health.

## Figures and Tables

**Table 1:** Signalling pathways involved in the regulation of adipogenesis.

Signalling Pathways	Effect on Adipocyte Differentiation	References
IGF-1	Promotes	[37,38,39,40]
Glucocorticoid	Promotes	[42,43,44,45]
cAMP	Promotes	[46,47,48,49,50]
TGF-β1 and 2	Inhibits	[52,53,54,55]
BMP2	Promotes	[59,60]
BMP4	Promotes	[53,56,57,58]
BMP7	Promotes	[55,61,62,63,64]
Wnt	Inhibits	[65,69,70,72]
Hedgehog	Inhibits	[74,76]
ERK/MAPK	Promotes	[78]
	Inhibits	[79,80]
P38/MAPK	Promotes	[81,82,83]
	Inhibits	[77]
Ras	Promotes	[37,84]
Retinoblastoma protein	Inhibits	[85]
Myostatin	Inhibits	[88]

**Table 2:** Epigenetic factors involved in the regulation of adipogenesis.

Regulator	Effect on Adipogenic Differentiation		References
Chromatin Remodelling Complex	In Vitro	In Vivo	
SWI/SNF	Promotes	-	[91]
**Lysine methyltransferases**			
SETDB1	Inhibits	-	[95,96]
G9a	Inhibits	Inhibits	[98]
Nsd2	Promotes	Promotes	[99]
MLL3/4	Promotes	Inhibits	[103]
Ezh2	Promotes	-	[94,102]
Ehmt1	-	Promotes	[104]
**Lysine demethylases**			
LSD1	Promotes	Promotes	[95,97]
Kdm5	Promotes	-	[100]
**Arginine methyltransferases**			
CARM1	Promotes	Promotes	[90,105]
PRMT5	Promotes	-	[106,107]
**Histone acetyltransferases**			
Gcn5/PCAF	Promotes	Promotes	[110]
CBP/p300	Promotes	Promotes	[111,112]
Epigenetic reader BRD4	Promotes	Promotes	[114,115]
**Histone deacetylases**			
HDAC 1	Inhibits	-	[117]
HDAC 1 and 2	Promotes	-	[119]
HDAC 9	Inhibits		[120]
Sirt 1	Inhibits	Inhibits	[123,124,132]
Sirt 2	Inhibits	-	[125]
Sirt 6	Promotes	Promotes	[126]
Sirt 7	Promotes	Promotes	[121]
**DNA methyltransferase**			
Dnmt1	Promotes (clonal expansion)	-	[129]
**DNA demethylases**			
Tet 1 and 2	Promotes	-	[131]

**Table 3:** MicroRNAs involved in the regulation of adipogenesis.

MicroRNAs	Target	Experimental Model	References
**Proadipogenic**			
MiR-143	ERK5 (MAPK signalling pathway)	Human preadipocytes	[135]
MiR 17-92	RB2/P130	3T3-L1 cells	[138]
MiR-125b-5p	Smad 4	3T3-L1 cells	[139]
MiR 30 a and d	Runx2	HASCs	[140]
MiR-204 and MiR-211	Runx2	C3H10T1/2	[141]
MiR-124	Dlx4	3T3-L1 cells	[136]
MiR-210	Tcf712 (Wnt signalling pathway)	3T3-L1 cells	[69]
MiR-146	Sirt 1/FOXO1	3T3-L1 cells	[144]
**Antiadipogenic**			
MiR-130	PPARγ	3T3-L1 cells	[142]
MiR-27a and b	PPARγ and C/EBPα	3T3-L1 cells	[143]
MiR-93	Sirt 7 and Tbx3	miR-25-93-106b–/– mice	[145]
